# Combining interictal intracranial EEG and fMRI to compute a dynamic resting-state index for surgical outcome validation

**DOI:** 10.3389/fnetp.2024.1491967

**Published:** 2025-01-28

**Authors:** Varina L. Boerwinkle, Kristin M. Gunnarsdottir, Bethany L. Sussman, Sarah N. Wyckoff, Emilio G. Cediel, Belfin Robinson, William R. Reuther, Aryan Kodali, Sridevi V. Sarma

**Affiliations:** ^1^ Division of Child Neurology, University of North Carolina in Chapel Hill, Chapel Hill, NC, United States; ^2^ Department of Biomedical Engineering, Johns Hopkins University, Baltimore, MD, United States; ^3^ Neuroscience Research, Barrow Neurological Institute at Phoenix Children’s Hospital, Phoenix, AZ, United States; ^4^ Brainbox Inc., Baltimore, MD, United States

**Keywords:** drug-resistant epilepsy, seizure onset zone, interictal intracranial EEG, resting-state fMRI, dynamic network modeling

## Abstract

**Introduction:**

Accurate localization of the seizure onset zone (SOZ) is critical for successful epilepsy surgery but remains challenging with current techniques. We developed a novel seizure onset network characterization tool that combines dynamic biomarkers of resting-state intracranial stereoelectroencephalography (rs-iEEG) and resting-state functional magnetic resonance imaging (rs-fMRI), vetted against surgical outcomes. This approach aims to reduce reliance on capturing seizures during invasive monitoring to pinpoint the SOZ.

**Methods:**

We computed the source-sink index (SSI) from rs-iEEG for all implanted regions and from rs-fMRI for regions identified as potential SOZs by noninvasive modalities. The SSI scores were evaluated in 17 pediatric drug-resistant epilepsy (DRE) patients (ages 3–15 years) by comparing outcomes classified as successful (Engel I or II) versus unsuccessful (Engel III or IV) at 1 year post-surgery.

**Results:**

Of 30 reviewed patients, 17 met the inclusion criteria. The combined dynamic index (im-DNM) integrating rs-iEEG and rs-fMRI significantly differentiated good (Engel I–II) from poor (Engel III–IV) surgical outcomes, outperforming the predictive accuracy of individual biomarkers from either modality alone.

**Conclusion:**

The combined dynamic network model demonstrated superior predictive performance than standalone rs-fMRI or rs-iEEG indices.

**Significance:**

By leveraging interictal data from two complementary modalities, this combined approach has the potential to improve epilepsy surgical outcomes, increase surgical candidacy, and reduce the duration of invasive monitoring.

## 1 Introduction

Epilepsy is a devastating neurological disease that affects more than 50 million people globally, according to the World Health Organization, with 30% of cases classified as drug-resistant epilepsy (DRE) (Kwan and Brodie, 2010; Kwan and Sander, 2004; Wieser et al., 2001). DRE causes significant costs, morbidity, and mortality (Engel, 2016; Laxer et al., 2014; Sillanpaa and Shinnar, 2010). The most effective treatment for DRE is surgery (Luders et al., 2006), which requires accurate localization of the seizure onset zone (SOZ) for success. Unfortunately, given the high diagnostic and surgical costs ($200,000/patient) (Begley et al., 2000; Murray et al., 1996), many opt out of this potentially curative procedure (England et al., 2012).

The first step in epilepsy surgery evaluation is a noninvasive multi-modality investigation, which may include anatomical magnetic resonance imaging (MRI), resting-state functional MRI (rs-fMRI), functional MRI (fMRI), scalp electroencephalography (EEG), simultaneous EEG-fMRI, positron emission tomography (PET), single-photon emission computed tomography (SPECT), and magnetoencephalography (MEG). Among the results, the clinically annotated SOZ-candidate (ca-SOZ) locations are often discordant. As surgery is typically permanent and has risks, if discordancy results in high uncertainty in the true location of the SOZ, then the ca-SOZ guides the placement of *invasive* intracranial EEG (iEEG) (Rosenow and Luders, 2001; Rosenow and Luders, 2001). iEEG is considered the gold standard of preoperative SOZ localization, yielding the highest assurance of localization of the “true positive” SOZ (tp-SOZ).

Despite the integration of noninvasive and invasive approaches for SOZ localization, surgical failure rates remain high, ranging from 30% to 70% (Bulacio et al., 2012; Engel, 2016; Gonzalez-Martinez et al., 2007; Laxer et al., 2014; Malmgren and Edelvik, 2017; McIntosh et al., 2004; Sillanpaa and Shinnar, 2010). Surgical failures may arise for several reasons, including mislocalization, inadequate resection (especially in proximity to functional areas), or secondary epileptogenesis. The proposed computational tool integrates advanced imaging biomarkers to address these challenges and enhance the accuracy of SOZ localization, thereby improving the prediction of surgical outcomes.

Unfortunately, iEEG is a time-intensive and resource-demanding procedure, as it requires clinicians to monitor patients until seizures occur (Engel, 2016; Laxer et al., 2014; Sillanpaa and Shinnar, 2010). Patients often remain in the epilepsy monitoring unit for 1–3 weeks, during which tens of seizures may be recorded, each of which may last a few minutes. To interpret this data, clinicians rely on stereoelectroencephalography (sEEG), a specific form of iEEG that uses depth electrodes to monitor brain activity. sEEG signals recorded immediately before, during, and after seizures are critical for localizing the SOZ, rendering most interictal recordings less actionable. Despite its utility, iEEG fails to localize the SOZ in 10%–15% of patients (MacDougall et al., 2009; Pondal-Sordo et al., 2007; Steriade et al., 2019). In such cases, only interictal information is available, further limiting the utility of iEEG in these patients. Given these limitations, novel approaches that focus on interictal data and leverage complementary modalities are critically needed.

To address this, we propose a novel approach that reduces the subjective interpretation of iEEG and rs-fMRI data by integrating these modalities into a unified framework. This method leverages the complementary strengths of both modalities to further enhance SOZ localization. Most importantly, it captures dynamic interictal signatures, an underutilized yet valuable source of information for SOZ localization. To reduce subjective interpretation, many proposed SOZ-localizing computational algorithms of fMRI (Rolls et al., 2020) and/or iEEG data (Burns et al., 2014; Gliske et al., 2016; Holler et al., 2018; Khambhati et al., 2015; Li et al., 2018; Yaffe et al., 2015) have yet to realize clinical impact. This may be due to epilepsy being a brain network disorder, and an important limitation of these computational approaches is that they either rely on the traditional *static* functional connectivity of networks, such as Pearson correlation analysis, and have thus ignored possibly key dynamic network properties, or they are too cumbersome because they require the capture of seizures. Furthermore, iEEG and rs-fMRI measure two different time scales of brain activity, thus possibly co-informing on the other’s “blind spots,” which may increase the sensitivity and specificity of SOZ detection and localization.

**FIGURE 1:**
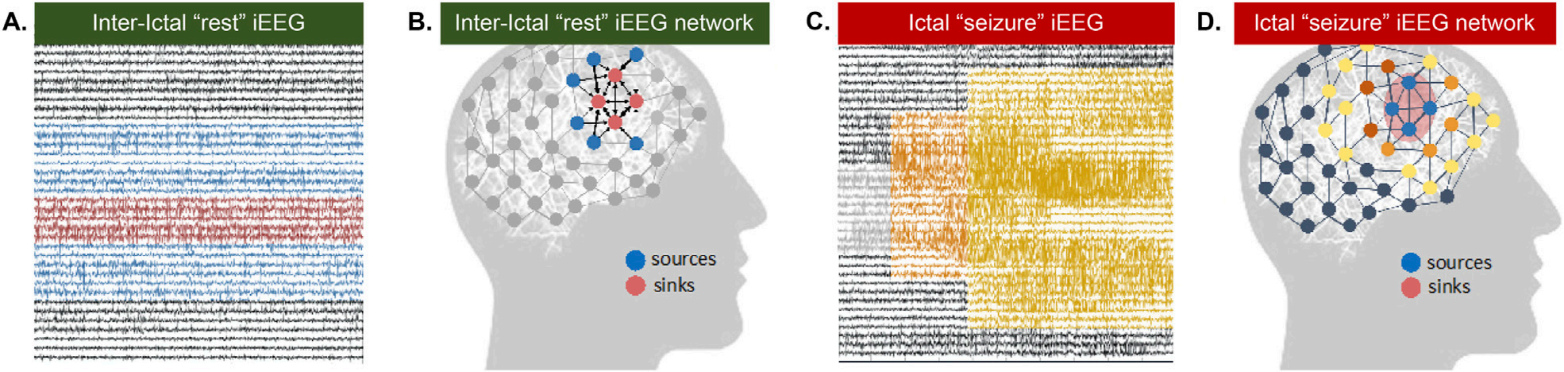
Source Sink Hypothesis. **(A)** Interictal (between seizure or at rest) iEEG snapshot and **(B)** corresponding source-sink schematic where sinks represent seizure focus **(C)** Ictal (seizure) EEG snapshot and **(D)** corresponding source-sink schematic where sources represent seizure focus.

To overcome the need for capturing seizures to “see” where they may originate, we propose leveraging connectivity properties derived from *dynamic* network models (DNMs) from the combination of synergistic rs-fMRI and interictal “resting-state” intracranial EEG (rs-iEEG) data. We posit that dynamic network information may help us “see” the SOZ by differentiating regions that exhibit excitation versus inhibition, which static techniques do not evaluate well. More specifically, we hypothesize that the interictal epileptic brain network is not seizing because neighboring regions are inhibiting the SOZ and that a seizure occurs when this inhibition temporarily becomes disabled (Figure 1). We further hypothesize that the maximal excitation comes from the SOZ toward these inhibiting regions, and this will add to the approach’s localization capacity.

Therefore, we propose to identify two groups of nodes in the brain network from rs-fMRI and rs-iEEG-derived DNMs: nodes that are inhibiting a specific group of nodes (sources) and the group of inhibited nodes themselves, that is, the SOZ (sinks) (Gunnarsdottir K. M. et al., 2022). Our source-sink hypothesis was validated in a retrospective study including 65 adult patients who underwent iEEG monitoring (Gunnarsdottir K. M. et al., 2022). The source-sink hypothesis is also supported by a recent study that demonstrated high inward-directed connectivity computed to the SOZ from rs-iEEG (Gunnarsdottir K. M. et al., 2022). However, this study relied on pre-selecting specific frequency bands to analyze and compute connectivity from static graph theoretic measures and could not reliably distinguish whether connections are excitatory or inhibitory. In rs-fMRI, we tested this hypothesis in a homogeneously located SOZ cohort that showed high SOZ concordance with surgical outcomes and did differentiate between excitation and inhibition (Boerwinkle V. L. et al., 2022). However, the population had subcortical SOZ from hypothalamic hamartoma; thus, this method has yet to be validated in cortical epilepsy, which has much greater variability in location and expected greater variability in network dynamics.

The aim of this study is to develop a comprehensive epilepsy surgery prediction tool by integrating SSIs of DNMs from both rs-iEEG and rs-fMRI and vet this against surgical outcomes. By relying solely on resting-state data, this approach has the potential to minimize the need for prolonged iEEG monitoring, reducing patient burden and improving surgical planning.

## 2 Methods

### 2.1 Study participants and overview of study workflow

This retrospective analysis was approved by the local institutional review board. Rs-fMRI became the standard evaluation for epilepsy surgery at Phoenix Children’s Hospital in May 2017, following evidence that surgical targeting of the rs-fMRI–detected SOZ improved surgical outcomes in pediatric epilepsy with a favorable safety profile (Boerwinkle V. L. et al., 2017). Rs-fMRI scans were obtained regardless of the timing of the last known seizure event, and no additional consent was required.

The retrospective collection of study data was conducted by reviewing the electronic medical record. The study included patients aged 6 months to 18 years diagnosed with drug-resistant epilepsy (DRE) under the care of the institutional epilepsy surgery team. Inclusion criteria were: preoperative continuous video iEEG and rs-fMRI with high-quality, artifact-free data obtained between May 2017 and December 2020; documented ca-SOZ prior to surgery; post-operative brain imaging; and seizure frequency documented preoperatively and at 1 year postoperatively. Surgical outcomes were classified using the Engel Epilepsy Surgery Outcome Scale (Engel, 1993): Engel class I and II outcomes were defined as successful (free of disabling seizures), while Engel classes III and IV were defined as failures.

**FIGURE 2:**
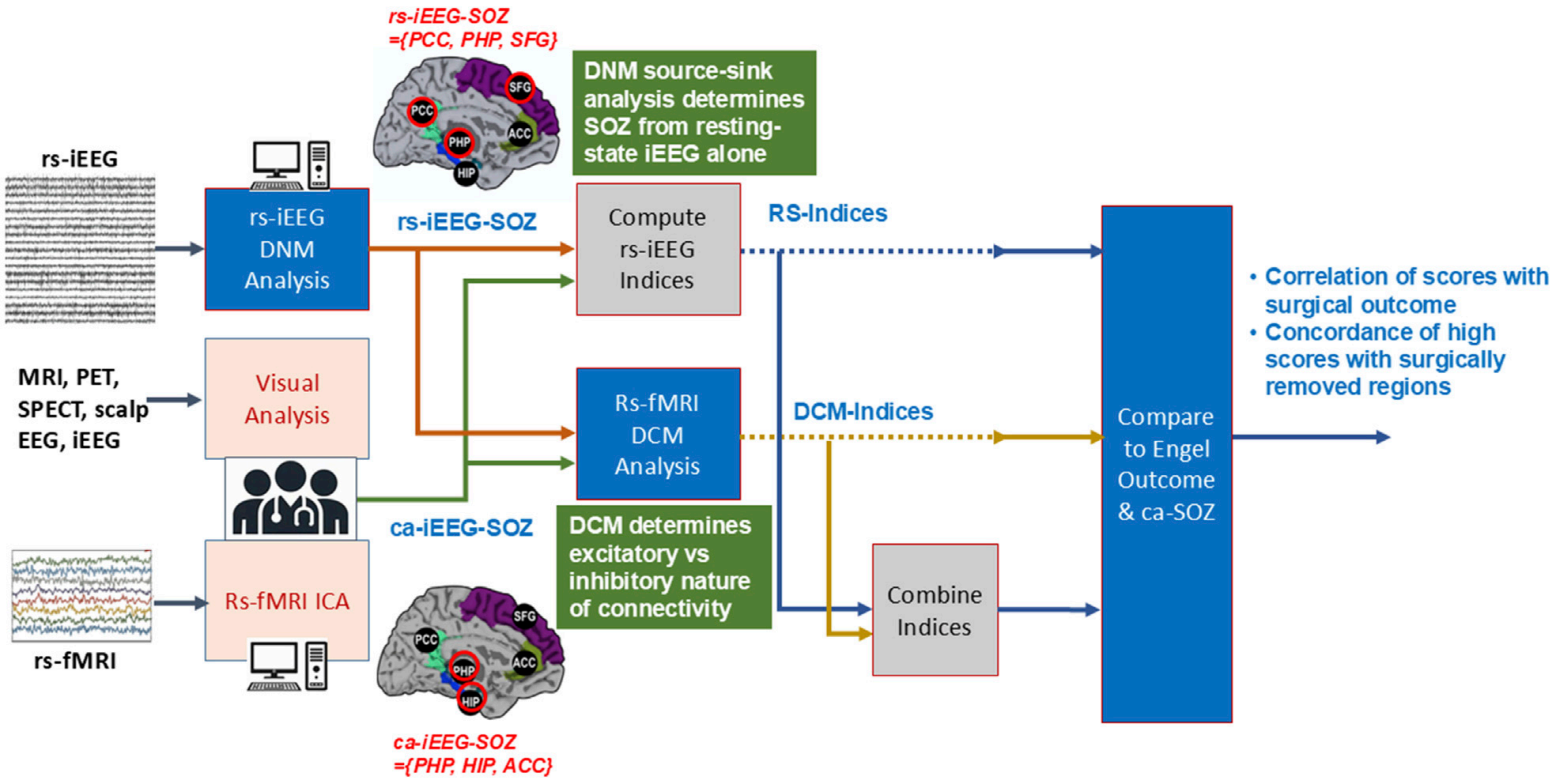
Workflow of study. Clinically annotated SOZ-candidate locations were derived from clinician interpretations of multiple noninvasive modalities, including MRI, EEG, and rs-fMRI by ICA. These informed the placement of the sEEG depth electrodes. The clinicians interpreted the sEEG and determined the ca-iEEG-SOZ. From the locations of the depth electrode placement, the rs-fMRI DCM, rs-iEEG DNM, and their combined dynamic indices were then determined. The index values were compared to the SOZ determined by clinicians’ sEEG interpretation and separately with the combination of surgical location and Engel outcomes.

**FIGURE 3:**
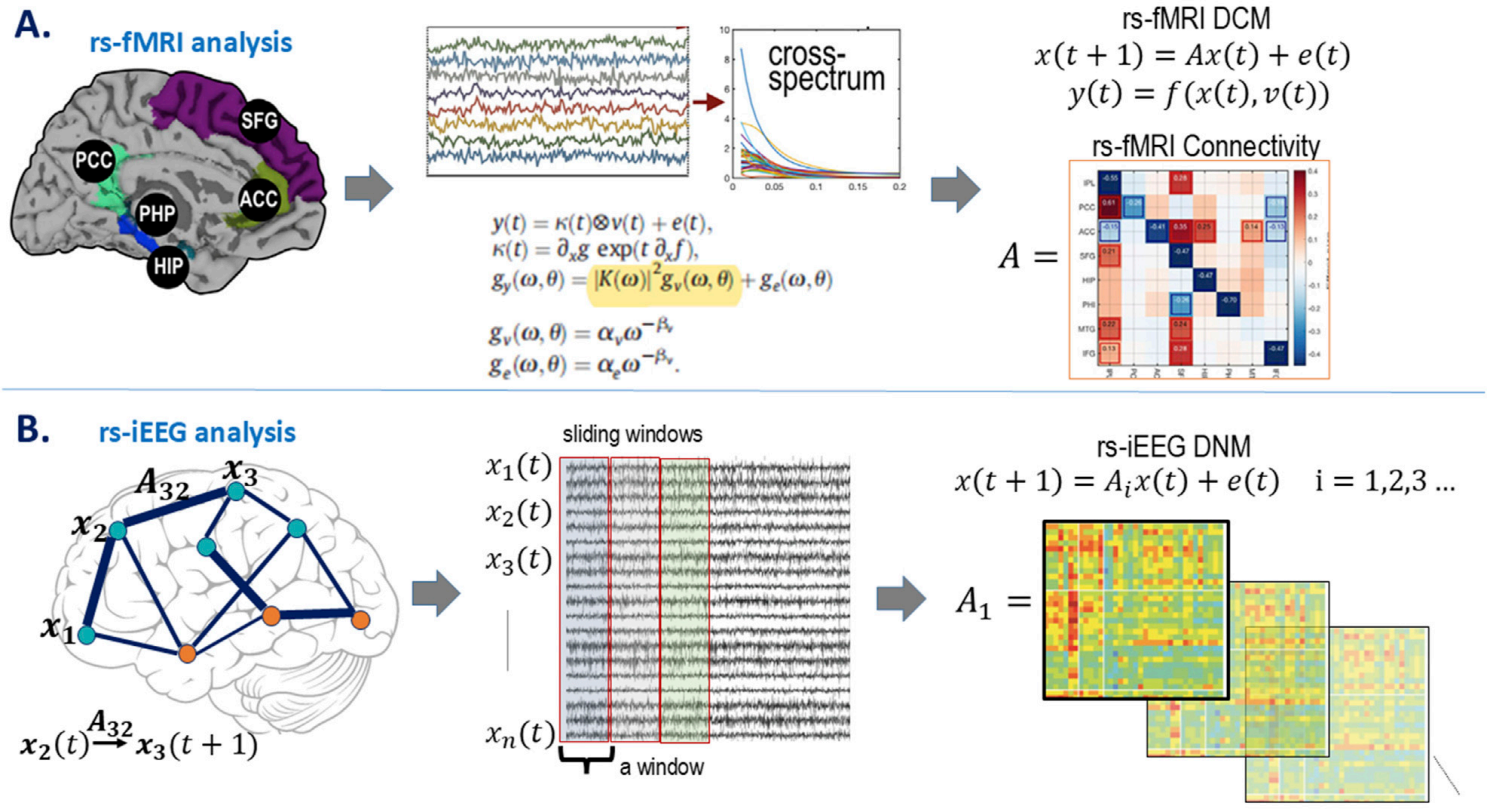
Model estimations. **(A)** rs-fMRI model estimation. Demonstrative node locations with the rs-fMRI BOLD signal over time are transformed from the time domain, $y\left(t\right)$ , to the spectral domain, where $K\left(\omega \right)$ is the Fourier transform of the system Volterra kernel $K\left(t\right)$ , which is a function of the effective connectivity ( A ). $g_{v}$ represents the effect of other nodes on a given region, $g_{e}$ is the endogenous effect of the region on itself, and $g_{y}$ is the cross-spectrum effective connectivity, taking both these exogenous and endogenous signals (amplitude ( α ) and slope ( β )) into consideration. **(B)** rs-iEEG model estimation. Model notation defined on electrode implantation. Each channel is a “node” in the iEEG network, where the signal on node i is denoted as $x_{i}\left(t\right)$ . The influence that node j has on node i is captured through $A_{ij}$ . The DNM, based on interictal iEEG, is characterized by the A matrix for each 500-ms window, generating a sequence of matrices resulting in one linear time-varying DNM.

An overview of the study workflow is presented in Figure 2. Rs-iEEG and rs-fMRI data were modeled using dynamic network analyses (Figure 3) and compared to the clinician-interpreted SOZ from iEEG and surgical outcomes. Both rs-fMRI and rs-iEEG analyses used sliding windows to transform interictal time series data into matrix-based connectivity values, which were then used to derive SSI scores.

### 2.2 Surgical outcomes and ROIs

Of the 30 patients reviewed, 17 consecutive patients met the study eligibility criteria, as detailed in Table 1. Of these 17, nine were female subjects, with an age range of 3–15 years (mean: 8.6 years). Reasons for exclusion, along with the number of patients excluded for each reason, were as follows: 1) Five patients lacked an adequately defined ca-SOZ due to non-localizing seizure activity; 2) one patient had corrupted sEEG lead data; 3) five patients underwent monitoring with grids and/or strips rather than sEEG; 4) Two patients had a ca-SOZ determined to be bilateral and diffuse across more than one lobe in a hemisphere and subsequently underwent bilateral recurrent nerve stimulator (RNS) placement. The epilepsy etiologies of the included patients were as follows: seven with focal cortical dysplasia or low-grade tumor, three with tuberous sclerosis (TSC), three with non-TSC brain malformations, two with MRI-negative epilepsy and no other identified etiology, one with mesial temporal sclerosis, and one with prior meningoencephalitis.

**TABLE 1:** Patient demographics, exclusion criteria, and surgical data.

ID	Included no.	Sex 0 = M; 1 = F	Age in years	Exclusion criteria^a^	1 = Laser; 2 = open craniotomy procedure; 3 = RNS	Lobe 1 = F; 2 = T; 3 = P, 4 = O, 5 = 2 or more lobes	Surgical location; 1 = R; 0 = L; 2 = B	Etiology code^b^	Engel outcome
A		0	5	1	2	1	1	1	IV
B		1	6	1	2	1	1	1	Ia
C		0	10	3	2	1	1	3	IV
D	1	1	8		2	1	0	3	II
E		1	12	3	2	2	0	1	III
F	2	1	13		2	5	1	1	II
G	3	1	6		1	1	1	3	Ia
H	4	0	5		2	2	1	1	IV
I	5	1	9		2	2	0	2	IV
J	6	0	11		2	2	1	1	Ia
K	7	0	7		2	2	1	1	1
L	8	1	4		1	5	0	1	1
M	9	0	3		1	1	0	2	1
N	10	1	12		2	1	0	1	II
O		1	10	3	3	1	1	7	III
P		0	7	3	2	1	1	0	I
Q		0	6	1	3	5	0	6	IV
R	11	0	5		1	1	0	2	IV
S		0	5	1	3	1	0	6	III
T	12	0	15		1	5	1	6	I-D
U		1	16	3					
V	13	1	13		1	2	1	4	IV
W		0	12	2					
X	14	1	9		2	2	1	3	I
Y		0	8	4	3	5	0	5	I
Z		1	14	1					
AA	15	0	8		1	1	0	1	III
BB	16	1	6		1	5	0	5	I
CC	17	0	12		3	5	2	5	II
DD		1	13	4	1	1	1	5	IV

^a^ Exclusion criteria: 1 = No ca-SOZ due to lack of localizing seizure activity during SEEG; 2 = SEEG but leads went bad before seizure captured, resulting in a poor quality SEEG; 3 = grid/strips with seizure; 4 = ca-SOZ, bilateral and diffuse beyond one lobe in each hemisphere.

^b^ Etiology code: 1 = FCD or low-grade tumor; 2 = TSC; 3 = non-TSC, congenital brain malformation; 4 = MTS; 5 = acquired brain insult including traumatic brain injury (TBI) or prior meningoencephalitis; 6 = MRI, negative; 7 = genetic.

The surgical procedure types were eight stereotactic laser ablations, eight open craniotomy approaches, and one RNS. The RNS procedure was performed for a patient whose SOZ was bilateral and diffuse, rendering resective or ablative surgery unsuitable. Although RNS is considered a palliative treatment, this case was included as the SOZ-candidate regions could still be evaluated using the same biomarker methods. Surgical locations were eight right, eight left, and one bilateral (RNS); of these, six were frontal, six were temporal, and five involved two or more lobes. The number of patients with 1-year Engel I, II, III, and IV outcomes was 8, 4, 1, and 4, respectively. The numbers of SOZ-candidate regions of interest (ROIs) per patient were one patient with two ROIs, seven patients with three ROIs, and eight patients with four ROIs. Three patients did not have post-operative imaging. Among the 17 patients, there were a total of 59 ROIs. Of these, 16 of 28 ca-ICA-SOZ overlapped the ca-SEEG-SOZ.

### 2.3 Data acquisition and preprocessing

*Rs-fMRI Acquisition and Preprocessing:* The rs-fMRI was performed as part of the standard presurgical evaluation on a 3-T Philips MRI with a 32-channel head coil. Conscious sedation was administered when clinically indicated, according to institutional standards, to ensure image quality. Patients were instructed to rest with their eyes closed if awake. Imaging parameters included T2*-weighted sequences with a TR of 2000 ms, TE of 30 ms, matrix size 80 × 80, and voxel dimensions of 3.4 × 3.4 × 3.4 mm³. Each session consisted of two 10-min runs (600 total volumes). Detailed parameters are provided in Supplementary Material Section 2.2.

Rs-fMRI preprocessing followed previously established clinical pipelines (Boerwinkle et al., 2019; Boerwinkle et al., 2018a; Boerwinkle et al., 2020; Boerwinkle V. L. et al., 2017; Boerwinkle et al., 2022a; Boerwinkle et al., 2022b; Boerwinkle et al., 2018b). In brief, data were high-pass filtered (cutoff = 100 s), spatially smoothed (1 mm), and realigned to the mean functional image. Functional scans were co-registered to the anatomical T1 image, visually inspected, and subjected to independent component analysis (ICA) using the FMRIB Software Library (FSL) tool MELODIC. ICA was applied to separate the Blood oxygenation level dependent (BOLD) signal into independent components generated by brain networks, which were evaluated for suspected SOZs based on validated spatial and temporal features (details in Supplementary Material, Section 2.2). The general linear model (GLM) was used to extract gray matter voxel time courses from predefined SOZ-candidate locations, excluding white matter, cerebrospinal fluid, and extracranial voxels.

*iEEG Acquisition and Preprocessing:* iEEG data were recorded using depth electrodes placed according to clinical indications. Data acquisition utilized the XLTEK EEG/Sleep System with a maximum sampling rate of 2000 Hz for intracranial electrodes. Following surgical placement, patients underwent continuous monitoring in the epilepsy monitoring unit for 1–21 days. Preprocessing included bandpass filtering (0.5–300 Hz) and notch filtering at 60 Hz (±2 Hz) to remove artifacts. Electrodes not recording from gray matter or deemed “bad” were excluded, and signals were re-referenced using a common average. Further details on preprocessing steps and instrumentation are provided in Supplementary Material, Section 2.2.

The study SOZ location subtypes: 1) preoperatively clinically annotated SOZ (ca-SOZ), 2) rs-iEEG, 3) rs-fMRI, 4) combined dynamic rs-iEEG and rs-fMRI, and 5) post-operative outcome.

#### 2.3.1 Clinically annotated SOZ

For each patient, the ca-SOZs were evaluated by clinical investigators based on comprehensive patient evaluation data obtained through an independent two-step procedure (noninvasive and invasive). The evaluation included regions suspected of being the SOZ based on findings from anatomical MRI, EEG, MEG, PET, SPECT, semiology, and neuropsychological testing. Noninvasive data were used to define the ca-SOZ hypothesis and guide the sEEG implantation strategy. Additionally, ICA data from the rs-fMRI (Chakraborty et al., 2020) informed the ca-SOZ candidates, distinct from the study’s rs-fMRI dynamic causal modeling (DCM) SOZ candidates.

iEEG signals were also used to anatomically define the ca-SOZs through expert clinician visual analysis, focusing on regions involved at seizure onset, typically characterized by low-voltage fast activity. During the multidisciplinary patient management conference, the clinical team formulated a hypothesis regarding the classification of each node (anatomical area recorded by a single electrode contact) in the epileptic network based on anatomic-electro-clinical correlations. Each node was assigned to one of the following categories:

a) ca-SOZ: Nodes exhibiting the earliest electrophysiological changes during an ictal event, generally preceding the clinical onset of seizures;
b) Propagation zone: Nodes involved at the time of the earliest clinical (semiological) manifestations during an ictal event; or
c) Other: Nodes not falling into the above categories.

The ca-SOZ nodes will be hereafter denoted as the ca-SOZ-candidate set. The region(s) among the ca-SOZ that underwent surgery are reported in the results.

#### 2.3.2 Rs-iEEG SOZ

##### 2.3.2.1 Background on sources and sinks in a rs-iEEG network

Recently, the source-sink metrics were proposed as a promising interictal marker of the SOZ (Gunnarsdottir K. M. et al., 2022). The metrics are derived based on the source-sink hypothesis, which states that SOZ regions, denoted as sinks, are persistently inhibited by neighboring regions (denoted as sources) during interictal periods to suppress seizures.

We modeled the iEEG data using all iEEG electrodes as a dynamic network that captures how every network node (iEEG contact) evolves and interacts with every other node dynamically (Gunnarsdottir K. M. et al., 2022). The interictal DNM is constructed by concatenating a sequence of linear time-invariant (LTI) DNMs defined in each sub-window of the data as

$$x\left(t+1\right)=Ax\left(t\right)+e\left(t\right),$$

where $x\left(t\right)\epsilon R^{n*1}$ represents the iEEG channel signals, $A\epsilon R^{N*N}$ is the state transition matrix, N is the number of iEEG channels, and $e\left(t\right)$ represents white Gaussian noise, uncorrelated with $x\left(t\right).$

For each patient, the DNMs were estimated in every sliding window of the iEEG data via least squares to obtain a sequence of connectivity matrices over time, $A_{j}\epsilon \left[1,2,\ldots ,T\right]$ , where T is the number of 500 msec windows. From this sequence of $A_{j}$ matrices, we computed one overall computational matrix, A , to represent the DNM for each patient as

$$A=\frac{1}{T}\;\sum_{j=1}^{T}abs\left(A_{j}\right).$$

Unlike rs-fMRI, rs-iEEG cannot distinguish between excitatory and inhibitory connections due to its lower spatial resolution. Thus, we take the absolute values of the connectivity matrices as the only information we can glean from the rs-iEEG $A_{j}$ matrices, indicating the strength of the connection between any two nodes, hereafter referred to as the *amount of influence* one node has on another. In A , row i represents the amount of influence all other nodes have on node i , and column j represents the amount of influence node j has on all other nodes in the network.

**FIGURE 4:**
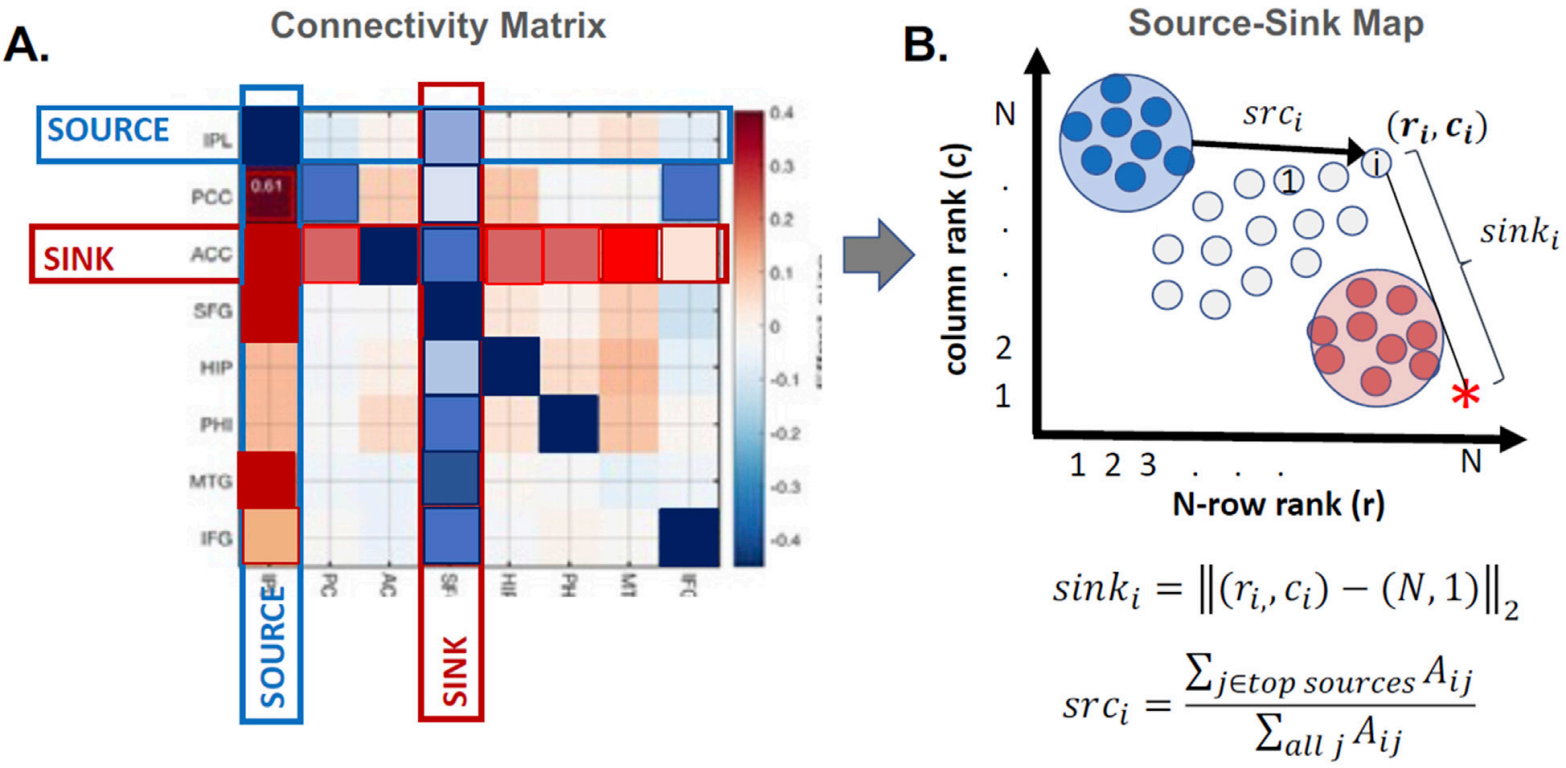
Patient-level source-sink algorithm. **(A)** Example of a connectivity matrix, A , derived from either the rs-fMRI DCM or rs-iEEG LTV DNM. Dark blue cells are significantly negative and represent inhibition, while dark red cells are significantly excitatory. If row j is relatively blue and column j is relatively red, node j received inhibition from other nodes in the network and projected excitation to other nodes in the network. **(B)** 2D source-sink map. Pink nodes represent top sinks, and blue nodes represent top sources.

Next, we identified the top sources and sinks of the iEEG network by quantifying the extent of each channel’s source and sink behavior and positioning them in a 2D source-sink space (Gunnarsdottir K. M. et al., 2022). Figure 4 shows a patient-level model of a 2D source-sink space. By definition, the top sinks of the iEEG network are in the bottom right, and the top sources are in the top left of the source-sink 2D space.

##### 2.3.2.2 Computing rs-iEEG SSI

Once all channels were plotted in the source-sink space, we computed four indices for each channel to quantify their hypothesized epileptogenicity. These indices are (Gunnarsdottir K. M. et al., 2022): a) sink index, which captures the distance from a given node to the ideal sink (Figure 4, pink star), b) source influence, which quantifies the amount of influence the node receives from top sources in the network, c) sink connectivity, which measures the influence from top sinks to the node, and finally, d) SSI, computed as the normalized (0–1 for each patient) product of the other three indices. The SSI will be high if all indices are high. We hypothesize that the larger the SSI for a given node, the more likely it is in the SOZ.

##### 2.3.2.3 Determining rs-iEEG SOZ candidates

In line with the assumption that the SOZ nodes are sinks inhibited by their neighboring sources at rest, the SSI score captures three criteria for SOZ nodes. First, a SOZ candidate must be a top sink, and second, it must be highly influenced (inhibited) by the top sources. Finally, the SOZ candidates are strongly connected to each other; that is, a SOZ node is also highly influenced by other top sinks.

For each patient, the LTI DNMs were constructed in every 500 msec sliding window of the iEEG data and then summarized by an overall connectivity matrix (Equation 2), which is depicted in Figure 4. From this matrix, an *SSI - EEG* score was obtained for each implanted iEEG channel. See Gunnarsdottir K. M. et al. (2022) for details on computing the SSI.

#### 2.3.3 Rs-fMRI DCM SOZ

##### 2.3.3.1 Background

DCM estimates neuronal interaction models to explain measured brain activity, as depicted in Figure 3 (Sussman et al., 2022). DCM uses differential equations to model brain responses generated by neural hemodynamic properties, providing Bayesian estimates of biologically related quantities, such as excitatory and inhibitory connectivity between neural populations. By modeling spectral dynamics, DCM evaluates how population interactions either increase or decrease each other’s activity over time.

Our application of DCM focuses on SOZ network directionality. It identifies regions with dominant inhibitory or excitatory connectivity, using Bayesian model averaging to optimize parameter estimates related to inhibition (Boerwinkle V. L. et al., 2022). Further details are provided in Supplementary Material, Section 2.2.3.

##### 2.3.3.2 Rs-fMRI preprocessing for DCM

The rs-fMRI data were pre-processed using an adapted SPM 12 pipeline (Friston et al., 1994), including slice timing correction, realignment, and co-registration to T1-weighted anatomical scans. A general linear model (GLM) was applied to denoise the data, removing contributions from white matter, cerebrospinal fluid, and motion-related noise. High-pass filtering excluded low-frequency artifacts, and data were not constrained to frequencies <0.1 Hz to capture potential SOZ-related activity (Boerwinkle V. L. et al., 2017). Further preprocessing details can be found in Supplementary Material Section 2.2.3.

##### 2.3.3.3 Defining rs-fMRI DCM SOZ-candidate ROIs

DCM “nodes” were defined as regions of interest (ROIs) likely to contain the SOZ (Supplementary Table S1). These ROIs were identified from multiple modalities, including:

a) rs-fMRI ICA candidate SOZs: Identified preoperatively by the surgical team based on clinical rs-fMRI findings;
b) ca-SOZ from ca-iEEG: Specified by clinical iEEG as part of the ca-SOZ;
c) Anatomical MRI lesions: Highlighted as probable SOZ regions. Notably, anatomical MRI regions often coincided with the ca-SOZ and were subsequently grouped with this category;
d) iEEG SSI score: Nodes surpassing a predefined threshold, signifying a SOZ, as indicated by interictal iEEG data from our collaborators.

Regions with substantial spatial overlap (>80% visual concurrence) were combined into a single ROI, while regions with less overlap were maintained separately. Specifically, ROIs were derived from:

1) The ca-SOZ from the ICA signal source mask;
2) The iEEG electrode locations encompassing the clinically annotated SOZ electrode/channels;
3) Anatomical lesion masks, such as hippocampal masks in mesial temporal lobe epilepsy.

ROIs were either manually drawn or created as spherical masks to cover suspected SOZ locations. For each ROI, the voxel time courses meeting a liberal threshold of <0.5 from the GLM were included in the computation of the first eigenvariate, which serves as a summary signal for the region. This threshold identifies voxels with statistically significant contributions to the first eigenvector, ensuring that the first eigenvariate captures the dominant temporal variation in the BOLD signal within the node. This approach minimizes the inclusion of irrelevant voxels and avoids relying on an unweighted mean of the ROI time series.

The rationale for including the candidate SOZ from option (d) above (iEEG SSI score surpassing the threshold) is supported by the source-sink methodology, recently published by the authors in a multicenter retrospective study. This method was shown to outperform high-frequency oscillations in identifying the SOZ (Gunnarsdottir et al., 2022). The source-sink methodology is grounded in a hypothesis about epileptic network properties during interictal periods: when the epileptic brain is not seizing, the SOZ is inhibited by other regions of the brain.

To test this hypothesis, we defined source nodes (influencers) and sink nodes (those being influenced) and developed a method to identify and quantify these nodes using iEEG and DNMs. Our study demonstrated a strong correspondence between the SOZ and sink nodes, providing support for the inhibition hypothesis. Unlike traditional connectivity measures such as functional connectivity, the SSI is derived from a dynamic network model, allowing it to capture the temporal interactions that underlie the inhibition phenomenon. Specifically, the SSI models how the activity of a given node at one time point influences the future activity of every other node in the network, thereby accounting for n-to-n dynamic interactions.

##### 2.3.3.4 DCM specification and estimation

DCM was implemented using the cross-spectral paradigm with DCM 12.5 in SPM (Friston et al., 2003; Friston et al., 2014). This approach models the spectral amplitude and phase of rs-fMRI data, deriving directional connectivity from auto- and cross-spectrum parameters (Friston et al., 2014). Fitting the connectivity model involves estimating parameters that describe the amplitude by frequency spectral representation for each region. Each region’s local spectrum is modeled as a power law distribution with an amplitude and scale, which indicates the frequency by amplitude slope. Directional connectivity is then derived by estimating the same parameters through frequency cross-spectrum between regions. DCM estimates parameters of auto- and cross-spectrum through multivariate auto-regression models of rs-fMRI’s BOLD data generating estimated spectrums (Friston et al., 2014).

For each subject’s initial model, we specified all possible connections as “on” and then inverted the model using cross-spectral DCM. We then performed a Bayesian model reduction that uses a greedy search to exhaustively permute the evidence for all possible variations of on/off connections within the full model (Friston and Penny, 2011). Finally, we used a Bayesian model averaging scheme that takes a maximum of the 256 models with the highest model evidence, weighs them by their model evidence, and averages the resulting parameters to produce an optimized model (Penny et al., 2010). By using Bayesian model reduction averaging over an exhaustive model space containing SOZ candidates, this generates parameter estimates to identify and link a region as a SOZ—the one that dominates in excitatory outbound connectivity. Importantly, this model averaging approach also provides a more agnostic single subject-based estimate to compare against other methods. Following Bayesian model averaging, the optimized parameters of the estimated connectivity matrix were in the log scale. The model did not include the diagonal, as self-modulation was not considered of interest. Scores were normalized from 0 to 1 per patient. These scores were derived from the corresponding A matrices (connectivity matrices) generated by their respective DNMs, including the source-sink index for rs-iEEG for both rs-fMRI and rs-iEEG.

#### 2.3.4 Post-operative outcomes

Post-operative outcomes were evaluated using the Engel Epilepsy Surgery Outcome Scale (Engel, 1993), which categorizes seizure outcomes at 1-year post-surgery. Successful surgical outcomes were defined as Engel Class I (seizure-free) or Class II (rare disabling seizures), while failure outcomes included Engel Class III (significant seizure reduction without seizure freedom) and Class IV (no improvement).

Seizure frequency and post-operative imaging data were documented during routine clinical follow-up at 3, 6, and 12 months post-surgery and abstracted from the electronic medical record by the study team. Post-operative brain imaging, when available, was reviewed to confirm the extent of resection or ablation and to ensure concordance with planned SOZ-targeted regions. For patients receiving palliative procedures (e.g., RNS), post-operative seizure frequency was assessed as part of standard care.

This data served as the ground truth for evaluating the predictive performance of the rs-iEEG and rs-fMRI-derived SOZ biomarkers, as well as the combined im-DNM index. Outcomes were compared against biomarker-detected SOZ candidates to validate their predictive accuracy.

#### 2.3.5 Combined dynamic iEEG-fMRI SOZ candidates

Finally, to further refine the hypothesized SOZ, we computed the combined iEEG-fMRI score, referred to as the dynamic index (im-DNM). The combined im-DNM is the average of the separate biomarker scores for each region. As rs-fMRI and iEEG dynamics measure different time scales, we expected these modalities to elucidate different aberrant network properties, which, when combined, may differentiate between those with projected good vs. poor outcomes for a given surgical plan. We evaluated separate and combined biomarker thresholds from 0 to 1 (see the index categorization below). We reported the separate and combined modality scores, thresholded to optimize the separate biomarker sensitivity and combined biomarker overall sensitivity and specificity.

### 2.4 Biomarker performance

To validate the biomarkers of DCM and rs-iEEG SOZs, we compared the biomarker scores across the planned ROIs to the surgical outcomes. Thus, patients were only counted once per person to reduce the variability of results due to the number of iEEG contacts and electrodes, which could skew the results. At a patient level, index scores for each modality (rs-iEEG, rs-fMRI, and combined rs-iEEG and rs-fMRI) were averaged across the ROIs for each patient. The distributions of rs-iEEG and rs-fMRI individual and combined SSI scores were evaluated in relation to successful outcomes (Engel I and II) and failed outcomes (Engel III and IV) for each modality. The threshold of each index was evaluated independently from each other between 0 and 1 by 0.05, with the optimal threshold selected based on peak performance in the separation of good from poor surgical outcomes of the respective indices.

## 3 Results

### 3.1 Combined DNM index scores across ROIs differentiate surgical outcomes

**FIGURE 5:**
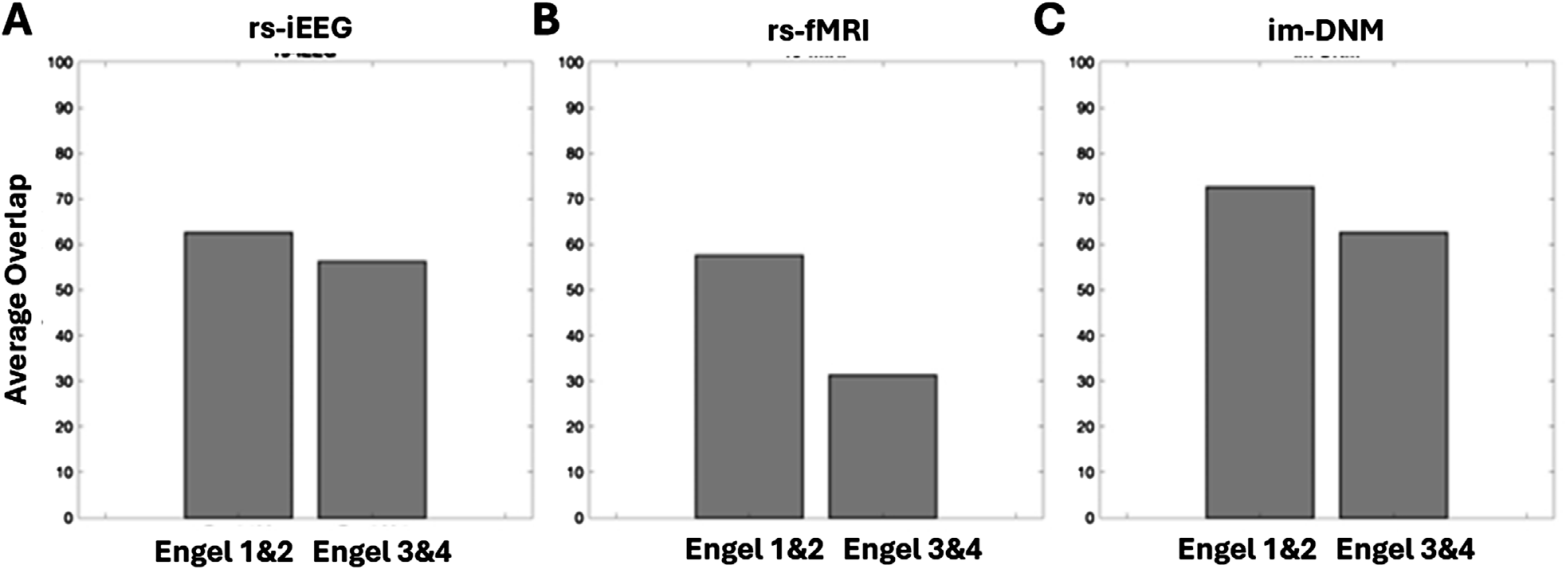
Score distributions of patient-level outcomes by biomarker subtype, showing **(A)** rs-iEEG, **(B)** rs-fMRI, and **(C)** the combined im-DNM with the optimal outcome group differentiation. The regions evaluated do not necessarily correspond with the surgically targeted region, as the goal was to evaluate regions with clinically reasoned SOZ hypothesis, and several patients had poor surgical outcome, implying the SOZ was outside the resected region.

Patient index scores for each modality (rs-iEEG, rs-fMRI) and for each ROI were computed, and their distributions are presented as box and whisker plots shown in Figure 5. Patient index scores for each modality were then averaged across the ROIs for each patient, and the combined score distributions are shown in Figure 5C. Figure 5 highlights that while both the rs-iEEG (Figure 5A) and rs-fMRI (Figure 5B) SSI scores show relatively equivalent differentiation between outcomes, the combined dynamic im-DNM index (Figure 5C) demonstrates superior performance, with no overlap between the successful and failed outcome groups.

### 3.2 DNM index scores for each modality may carry different information about SOZs

**FIGURE 6:**
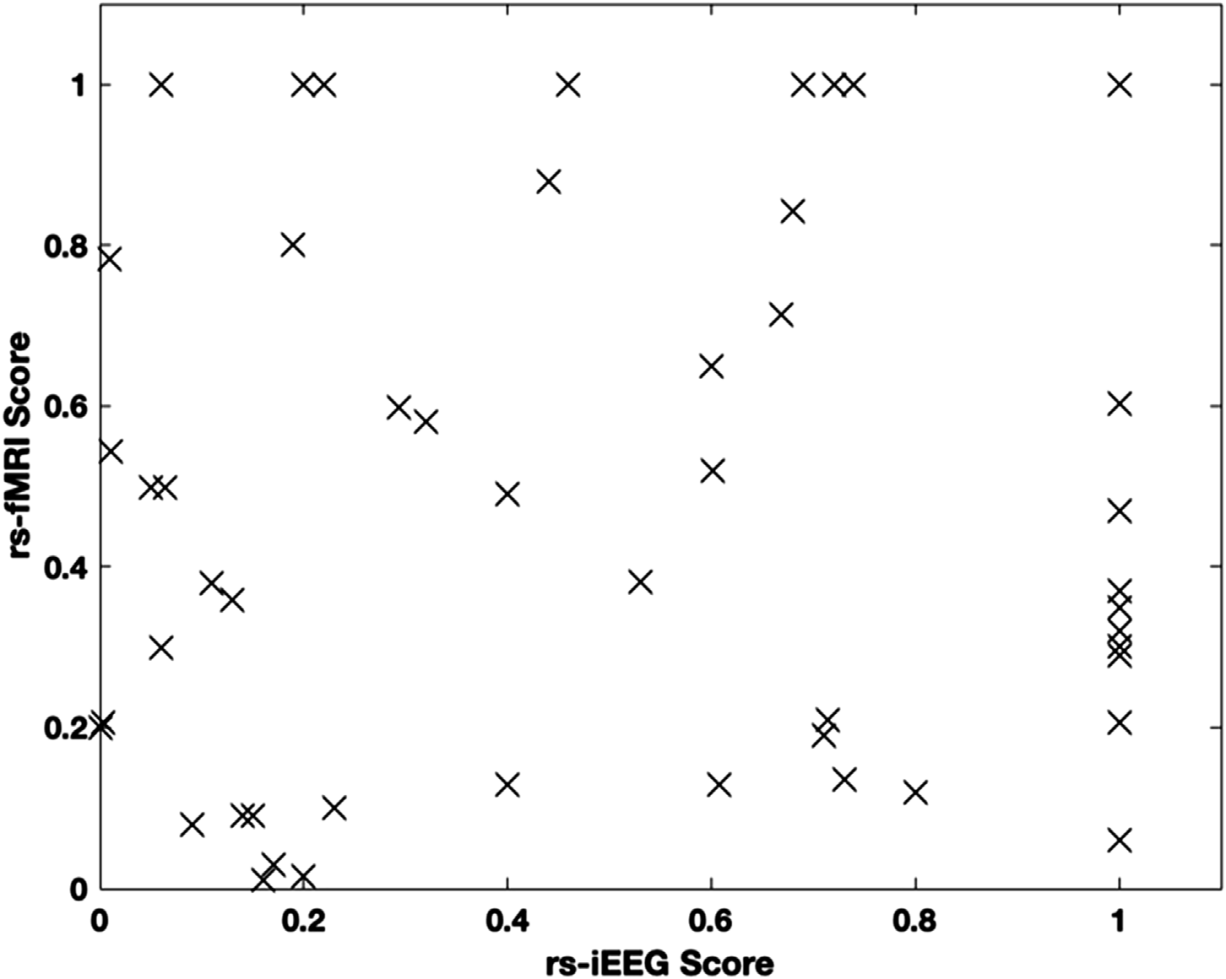
Scatterplot of each modality score.

These results suggest that the individual scores are biomarkers that carry different information about the SOZ. When plotted against each other, the patient scores for each modality are linearly correlated (rho = 0.26, p-value = 0.049). However, as shown in Figure 6, the correlation is not strong.

**FIGURE 7:**
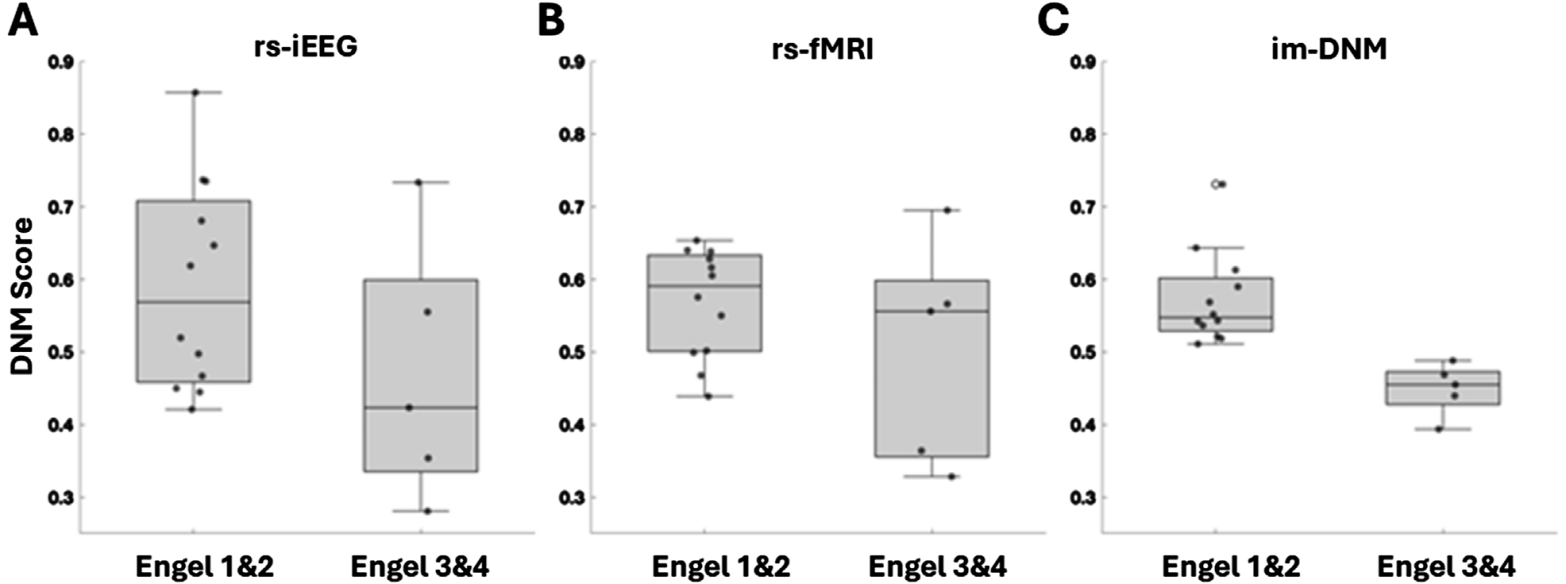
Comparison of index threshold values for differentiating good vs. poor outcome groups. Left to right are **(A)** rs-iEEG, **(B)** rs-fMRI, and **(C)** combined rs-iEEG and rs-fMRI as im-DNM index results categorized by the patient-level good vs. poor surgical outcomes on the *x*-axis. The bar graphs compare the index threshold at 0.9 via the percentage of overlap of patients’ thresholded index values with good vs. poor outcomes.

Figure 7 plots the percentage of ROIs, as determined by regions whose scores are greater than 0.9, that have at least 80% overlap with the surgically removed regions. All three scores show greater overlap with surgically removed regions in patients with good (Engel I and II) outcomes than in patients with bad outcomes. The rs-iEEG marker overlaps with 62% of surgically removed regions for good outcomes and 56% for bad outcomes. The rs-fMRI marker overlaps with 58% of surgically removed regions for good outcomes and 32% for bad outcomes. Finally, the combined marker overlaps with 72% of surgically removed regions for good outcomes and 63% for bad outcomes. The increased overlap with surgically removed ROIs in the combined marker for all outcomes, especially good outcomes, further supports the separation seen between good and bad outcomes in Figure 5C.

## 4 Discussion

The concept of combining indices from two different physiological measures of rs-iEEG and rs-fMRI on an individual basis to prospectively identify SOZ is novel. In this cohort of 17 patients with quality iEEG data, surgical outcomes, and rs-fMRI, the prediction of surgical outcomes improved when the thresholded index values of rs-iEEG and rs-fMRI were integrated into a combined index.

The improved performance of the combined measure may stem from several physiologically relevant factors. First, the rs-iEEG and rs-fMRI indices are sensitive and specific to different aspects of epileptogenic sources and sinks. Rs-iEEG offers millisecond-level temporal precision, whereas rs-fMRI provides whole-brain spatial coverage. Second, rs-iEEG captures electrical impulses from neuronal charge potentials across brain regions, while rs-fMRI reflects indirect oxygen utilization associated with neuronal activity within brain networks. These distinct physiological underpinnings allow the two modalities to capture complementary aspects of epileptogenic tissue. Importantly, the combined index enhances the ability to predict surgical outcomes based on biomarker evaluation of SOZ-candidate regions.

High-frequency oscillations (HFOs) are among the most extensively studied interictal iEEG features for identifying the epileptogenic zone (Gliske et al., 2016; Murphy et al., 2017; Akiyama et al., 2011; Wang et al., 2013; Bragin et al., 2010). Although regions within the epileptogenic zone often exhibit elevated HFO rates (Akiyama et al., 2011; Jacobs et al., 2010; Zijlmans et al., 2012), significant variability and debate persist regarding their reliability and predictive value for surgical outcomes. Two meta-analyses concluded that HFOs have limited predictive strength, with findings inconsistent across studies (Jacobs et al., 2018; van’t Klooster et al., 2017). Further challenges include variability in HFO definitions, observations in non-epileptogenic regions, and significant temporal instability in HFO rates, as demonstrated by Gliske et al. (2018), who found inconsistent identification of HFO channels across short recording segments.

These limitations underscore the need for biomarkers that are both robust and stable. Our combined DNM marker addresses these challenges by integrating source-sink analysis from rs-iEEG and dynamic connectivity modeling from rs-fMRI. Unlike HFOs, our approach demonstrated stability and reproducibility across random selections of interictal activity and was robust to the inclusion or exclusion of artifacts. This suggests that the combined biomarker may offer a more consistent and reliable predictor of SOZ localization and surgical outcomes, further advancing preoperative evaluation tools in epilepsy surgery.

The complexities of epilepsy surgery evaluation often make surgical decision-making far from straightforward. Thus, the proposed combined score provides surgeons with a tool to evaluate their surgical plan. A low combined score for a given surgical plan may flag a high likelihood of failure, whereas a high score indicates a greater likelihood of success. The ability to identify potential failures preoperatively is perhaps the most important utility of this combined score.

This dataset focuses on the pediatric DRE population, which arguably exhibits greater heterogeneity in SOZ location than the adult population, where mesial temporal SOZs are more common. Thus, we anticipate the performance of the biomarker will improve in adult populations wherein the ROIs are more likely to be localized, potentially increasing specificity. Future trials with adult populations are warranted to validate these findings and further explore the utility of this combined biomarker approach.

## 5 Limitations

As with all retrospective biomarker studies, fitting thresholds to optimize separation between good and poor outcomes can result in overfitting the data. To address this limitation, larger prospective studies with pre-determined thresholds are necessary to further validate these measures.

Surgical border-zone epileptogenic activity may influence post-operative seizure frequency, potentially impacting the observed outcomes. To better evaluate this factor, our study incorporates patient-level post-operative imaging and clinical condition data, which will be further analyzed in the ongoing full study.

Another limitation is the dependency of iEEG on prior SOZ location hypotheses generated from noninvasive tests. If the true SOZ does not adequately overlap with iEEG electrode placement, the resulting data may be low yield or misleading.

Finally, this study included only five patients in the Engel III–IV groups, which may skew the results and limit statistical power. This underscores the need for larger studies to validate the findings and ensure generalizability.

## 6 Conclusion

This study demonstrates that combining rs-fMRI and rs-iEEG indices in the im-DNM shows promise for improving epilepsy surgery outcome prediction compared to using either modality alone. Our findings, validated against surgical outcomes in this retrospective cohort, highlight the potential utility of this combined approach for preoperative SOZ localization.

However, these results are preliminary and limited to the analyzed dataset. Larger, prospective studies with independent validation cohorts are needed to confirm the predictive value of this combined biomarker and establish its clinical utility across diverse epilepsy populations. Future work should focus on refining the combined index and testing it on new patient datasets to further evaluate its generalizability.

## Data Availability

The raw data supporting the conclusions of this article will be made available by the authors, without undue reservation.

## Glossary

AAL3: Automated Anatomical Atlas 3
ACC: anterior cingulate cortex
AUC: area under the curve
BOLD: blood oxygenation level dependent
ca-SOZ: clinically annotated seizure onset zone candidate
CAT: computational anatomy toolbox
CSF: cerebrospinal fluid
DCM: dynamic causal modeling
DNM: dynamic network model
DRE: drug-resistant epilepsy
EEG: electroencephalography
GLM: general linear model
HFO: high-frequency oscillation
HIP: hippocampus
ICA: independent component analysis
iEEG: intracranial electroencephalography
IEDs: interictal epileptiform discharges
IFG: inferior frontal gyrus
im-DNM: dynamic index
IPL: inferior parietal lobe
LTI: linear time-invariant
MEG: magnetoencephalography
MRI: magnetic resonance imaging
MTG: middle temporal gyrus
PCC: posterior cingulate cortex
PET: positron emission tomography
PHP: parahippocampal gyrus
RNS: responsive neurostimulator
rs: resting state
rs-fMRI: resting-state functional magnetic resonance imaging
RSN: resting-state networks
rs-iEEG: resting-state intracranial stereoencephalography
sEEG: stereoelectroencephalography
SFG: superior frontal gyrus
SOZ: seizure onset zone
SPECT: single-photon emission computed tomography
SPM: statistical parametric mapping
SSI: source–sink index
tp-SOZ: true positive seizure onset zone
TSC: tuberous sclerosis
