# Supplementary material for: Combining interictal intracranial EEG and fMRI to compute a dynamic resting-state index for surgical outcome validation
Source: Front Netw Physiol. 2025 Jan 28;4:1491967. doi: 10.3389/fnetp.2024.1491967 (PMC11811083; doi:10.3389/fnetp.2024.1491967)
Supplement: Supplementary file 1 [file DataSheet1.docx]

Supplementary Material

Supplementary materials

2.2 Data Acquisition and Preprocessing

Original text:

Studies show that the impact on rs-fMRI functional connectivity from sedative agents is dose dependent (Grandjean et al., 2014; Liu et al., 2017; Metwali et al., 2019). High dose sedation is called general anesthesia and is used for invasive surgeries. General anesthesia results in lowering of brain network activity such that the patient is rendered unconscious and the impact on measured functional connectivity while the sedation is administered is substantial. Alternately, low dosage of sedative is called conscious or light sedation and the while there are some differences in functional connectivity (FC), it is substantially less than general anesthesia, as evidence by both the patient remaining conscious, and the FC reduction in the mild range, comparatively, as shown in Figure 1 of Liu et al. (Liu et al., 2017). Our center has interpreted over 2,500 individual rs-fMRI for primarily the purpose of epilepsy surgery evaluation. Most of these patients were children who were determined by their clinical team to need light conscious sedation for successful MRI scan acquisition. No safety events occurred during these scans and the quality of the images were evaluated by the clinical expert team on an individual basis. The results were found to be in line with the expected results such that the expected resting state networks (RSN) were all detectable in the expected locations including motor, language, default mode, fronto-parietal, vision, subcortical, and others. The location of the SOZ networks were compared to those detected by other modalities including iEEG, if available, and the ultimate surgical outcome in terms of seizure frequency (Boerwinkle et al., 2019; Boerwinkle et al., 2018a; Boerwinkle et al., 2020; Boerwinkle V. L. et al., 2017; Boerwinkle Varina L. et al., 2022a; Boerwinkle Varina L. et al., 2022b; Boerwinkle et al., 2018b). Thus, while the impact of the light conscious sedation is not zero on FC, it is low enough that the expected RSN and seizure networks were detected, in line with the current literature on this dose-dependent effect.

Awake patients were told to rest with their eyes closed. The rs-MRI sequence was: T2*- weighted images, TR (repetition time) 2000 ms, TE (echo time) 30 ms, matrix size 80 x 80, flip angle 80, number of slices to cover at least supratentorium, slice thickness such that voxel size is 3.4 x 3.4 x 3.4 mm3, no slice gap, inter-leaved top-down acquisition. The number of total volumes was 600, split between two equally timed runs, each equal to 10 minutes.

We applied the same processing steps that we have utilized clinically (Boerwinkle et al., 2019; Boerwinkle et al., 2018a; Boerwinkle et al., 2020; Boerwinkle V. L. et al., 2017; Boerwinkle Varina L. et al., 2022a; Boerwinkle Varina L. et al., 2022b; Boerwinkle et al., 2018b). Rs-fMRI preprocessing included a high-pass filter at 100s, spatial smoothing at 1 millimeter, and with motion corrected (Jenkinson et al., 2002). Preprocessing includes scan realignment to the mean functional image and then correcting for differences in timing. Aligned functional scans are then co-registered with the subject’s functional anatomical scan to ensure overlapping identification of brain regions, visually inspected by an expert. Afterward, the data underwent subject-level spatial independent component analysis (ICA) with FMRIB Software Library (FSL) tool MELODIC (Administration, 1988; Beckmann et al., 2005). As such, individual preprocessed blood-oxygen-level-dependent (BOLD) sequences underwent multi-session temporal concatenation. The resulting components were modeled through an automated dimensionality estimate using a Bayesian approach. ICA is a data-driven mathematical process that analyzes the BOLD signal, separating it into detected sub-signals called independent components generated by brain networks. Each brain network is distinguishable because it fluctuates in its oxygen concentration independently from the other brain networks, resulting from neural demands (Boerwinkle et al., 2016).

The clinical SOZ from rs-fMRI are determined using expert-based validated characteristics that differentiate them from noise and RSNs, such as the default mode network (Banerjee et al., 2023; Boerwinkle et al., 2019; Boerwinkle et al., 2018a; Boerwinkle Varina L. et al., 2017). This method has been validated through both comparison to seizure onset location by clinical intracranial EEG and the surgical outcomes. The characteristics include spatial and temporal features such as BOLD frequencies greater than those typically detected in RSN and being primarily localized in the grey matter and not conforming to the reliable spatial patterns of the RSN. Importantly, SOZ can spatially overlap with RSN, like how different RSN themselves can also spatially overlap. Thus, differentiating SOZ from RSN through the data driven method of ICA allows visualization of these networks in context of each other for the clinical team to appreciate the risks of permanently destroying a portion of the brain and what morbidity may be incurred through concurrent damage to other overlapping brain networks. The net result has been a reduction in surgical morbidity from more informed surgical planning.

Activity from rs-fMRI ICA-filtered data was extracted using a general linear model (GLM), not to be confused with the generalized linear model, from the predefined SOZ candidate locations. These locations were the union of the ca-SOZ and rs-iEEG-SOZ candidates, herein termed nodes in the SOZ network. The GLM step provided two simultaneous pre-processing components. First, it high-pass filtered the data to remove high-frequency non-neural artifacts from the data. Then, it extracted the gray matter voxel time-course across all scans while removing voxels in white matter, cerebrospinal fluid, or cranial cavity region while also correcting for subject movement that co-occurs with fMRI activation.

The iEEG included sEEG depth electrodes placed according to clinical needs determined by each patient’s expert care team. The data were recorded using the XLTEK System EEG/Sleep Acquisition with Neuroworks and Sleepworks software version 8.5. The EEG/Sleep amplifier utilized was the Brain Monitor Amplifier, with the breakout box “Connex” box. The max sampling rate was 2000 Hz for intracranial and 200 Hz for scalp EEG, and the system included a 256-channel box. After surgical placement, participants stayed in the inpatient epilepsy monitoring unit and were typically monitored between 1-21 days, depending on the clinical course.

First, sEEG contacts not recording from gray matter (e.g., white matter signals, outside of the brain, electrocardiogram signals, or reference signals) or otherwise deemed “bad” (e.g., broken or excessively noisy) by visual inspection were discarded from each patient’s dataset. Then, the data were bandpass filtered between 0.5 and 300 Hz with a fourth-order Butterworth filter, and notch filtered at 60 Hz (and its higher resonance frequencies) with a bandwidth region of 2 Hz. A common average reference was applied to remove common noise from the signals.

Revised text:

**Rs-fMRI Acquisition and Preprocessing:** Detailed sedation protocols were applied according to institutional standards. Light sedation was used for children requiring assistance to remain still during MRI acquisition, minimizing its impact on functional connectivity as supported by prior literature (Grandjean et al., 2014; Liu et al., 2017). Quality assurance confirmed detectability of resting-state networks (RSNs), including motor, language, default mode, and subcortical networks, ensuring data reliability for SOZ evaluation.

**Detailed Imaging Parameters:**

- Sequence Type: T2*-weighted images
- TR: 2000 ms
- TE: 30 ms
- Matrix Size: 80 × 80
- Voxel Dimensions: 3.4 × 3.4 × 3.4 mm³
- Slices: Sufficient to cover the supratentorium with no slice gap
- Acquisition Mode: Interleaved, top-down

**Detailed Preprocessing Steps:**

- Realignment of functional scans to the mean functional image for motion correction.
- Co-registration to T1-weighted anatomical images with visual inspection by experts.
- Temporal concatenation across runs for ICA, with Bayesian dimensionality estimation to identify independent components.

**iEEG Acquisition and Preprocessing:** The iEEG signals were acquired using a 256-channel EEG system with the XLTEK Brain Monitor Amplifier. Electrodes were placed according to the clinical hypothesis of SOZ location. Preprocessing steps were:

- Visual inspection to exclude non-gray matter or noisy electrodes.
- Bandpass filtering between 0.5 and 300 Hz using a fourth-order Butterworth filter.
- Notch filtering at 60 Hz and harmonics to remove electrical noise.
- Re-referencing to a common average to reduce noise.

**Section 2.2.3: Rs-fMRI DCM SOZ**

*Background on DCM:* DCM estimates neuronal interaction models by combining principles of control theory, differential equations, and Bayesian inference. The primary goal is to model the brain’s neuronal dynamics that give rise to observed BOLD time series data. DCM uses differential equations to capture how neuronal populations influence each other’s activity, integrating hemodynamic responses to estimate connectivity parameters.

In cross-spectral DCM, steady-state spectral representations of neuronal activity are derived via Fourier transformation. The spectral amplitude and phase characterize the dynamics of neuronal populations, enabling the estimation of directional connectivity (Friston et al., 2014). The parameters include:

- Auto-spectrum: Describes the power distribution within a node across frequencies.
- Cross-spectrum: Captures the relationship between spectral amplitudes of two nodes, reflecting directional connectivity.

*Rs-fMRI Preprocessing for DCM:* Rs-fMRI data preprocessing was performed using an adapted SPM12 pipeline. The steps included:

- Slice Timing Correction: Adjusted for interleaved slice acquisition timing differences.
- Realignment: Aligned functional scans to the mean image for motion correction.
- Co-registration: Registered functional scans to T1-weighted anatomical images.
- Denoising: Applied a general linear model (GLM) to remove signal contributions from white matter, cerebrospinal fluid, and six motion parameters.
- High-Pass Filtering: Removed low-frequency noise components (<0.01 Hz).

Preprocessing excluded spatial smoothing to preserve spatial specificity for DCM analysis. Functional images were normalized to MNI space using CAT12 deformation fields. To define anatomically informed regions of interest, the AAL3 atlas (Rolls et al., 2020) was mapped to native T1 space.

*ROI Definitions and Criteria:* To define DCM "nodes," regions of interest (ROIs) were identified using the following criteria:

- rs-fMRI ICA candidate SOZs: Identified by clinical experts pre-operatively.
- ca-SOZ from iEEG: Specified from clinical iEEG findings.
- Anatomical MRI lesions: Highlighted by anatomical MRI, often overlapping with ca-SOZ regions.
- iEEG SSI scores: Regions surpassing predefined thresholds for source-sink behavior.

Regions with >80% spatial overlap between modalities were merged into a single ROI, while less overlapping regions were treated as distinct but overlapping ROIs. ROIs were manually drawn or created as spherical masks centered on the suspected SOZ. For each ROI, voxel time courses contributing to the first eigenvariate were extracted using a liberal threshold of 𝑝 < 0.5. This eigenvariate captured the dominant variation of BOLD activity within the node.

*Bayesian Model Reduction and Averaging:* DCM specification included all potential connections between nodes in an initial full model. Parameters were then estimated using Bayesian model reduction (Friston and Penny, 2011), which iteratively tested all possible configurations of connectivity ("on/off" states) within the full model to maximize model evidence.

The final model was optimized using Bayesian model averaging (Penny et al., 2010):

- The 256 models with the highest model evidence were identified.
- Model parameters were weighted by their respective model evidence.
- Weighted averages of the parameters were computed to generate a single optimized connectivity matrix.

The model provided estimates of directional connectivity, identifying SOZ nodes as those with dominant excitatory outbound connectivity. Connectivity matrix parameters were log-transformed and normalized to a 0–1 range for patient-level comparison.

# 1 Supplementary Figures and Tables

**1.1**  **Supplementary Tables**

| **Table 1.** Patient Demographics, Exclusion Criteria, and Surgical Data | | | | | | | | | |
| --- | --- | --- | --- | --- | --- | --- | --- | --- | --- |
| ID | Included No. | sex 0=M; 1=F | age in years | Exclusion Criteria* | 1=Laser; 2=open craniotomy procedure; 3=RSN | lobe 1=F; 2=T; 3=P, 4=O, 5=2 or more lobes | surgical location; 1=R; 0=L; 2=B | Etiology Code** | Engel outcome |
| A |  | 0 | 5 | 1 | 2 | 1 | 1 | 1 | IV |
| B |  | 1 | 6 | 1 | 2 | 1 | 1 | 1 | Ia |
| C |  | 0 | 10 | 3 | 2 | 1 | 1 | 3 | IV |
| D | 1 | 1 | 8 |  | 2 | 1 | 0 | 3 | II |
| E |  | 1 | 12 | 3 | 2 | 2 | 0 | 1 | III |
| F | 2 | 1 | 13 |  | 2 | 5 | 1 | 1 | II |
| G | 3 | 1 | 6 |  | 1 | 1 | 1 | 3 | Ia |
| H | 4 | 0 | 5 |  | 2 | 2 | 1 | 1 | IV |
| I | 5 | 1 | 9 |  | 2 | 2 | 0 | 2 | IV |
| J | 6 | 0 | 11 |  | 2 | 2 | 1 | 1 | Ia |
| K | 7 | 0 | 7 |  | 2 | 2 | 1 | 1 | 1 |
| L | 8 | 1 | 4 |  | 1 | 5 | 0 | 1 | 1 |
| M | 9 | 0 | 3 |  | 1 | 1 | 0 | 2 | 1 |
| N | 10 | 1 | 12 |  | 2 | 1 | 0 | 1 | II |
| O |  | 1 | 10 | 3 | 3 | 1 | 1 | 7 | III |
| P |  | 0 | 7 | 3 | 2 | 1 | 1 | 0 | I |
| Q |  | 0 | 6 | 1 | 3 | 5 | 0 | 6 | IV |
| R | 11 | 0 | 5 |  | 1 | 1 | 0 | 2 | IV |
| S |  | 0 | 5 | 1 | 3 | 1 | 0 | 6 | III |
| T | 12 | 0 | 15 |  | 1 | 5 | 1 | 6 | I-D |
| U |  | 1 | 16 | 3 |  |  |  |  |  |
| V | 13 | 1 | 13 |  | 1 | 2 | 1 | 4 | IV |
| W |  | 0 | 12 | 2 |  |  |  |  |  |
| X | 14 | 1 | 9 |  | 2 | 2 | 1 | 3 | I |
| Y |  | 0 | 8 | 4 | 3 | 5 | 0 | 5 | I |
| Z |  | 1 | 14 | 1 |  |  |  |  |  |
| AA | 15 | 0 | 8 |  | 1 | 1 | 0 | 1 | III |
| BB | 16 | 1 | 6 |  | 1 | 5 | 0 | 5 | I |
| CC | 17 | 0 | 12 |  | 3 | 5 | 2 | 5 | II |
| DD |  | 1 | 13 | 4 | 1 | 1 | 1 | 5 | IV |

 *Exclusion Criteria: 1=No ca-SOZ due to lack of localizing seizure activity during SEEG;  2=SEEG but leads went bad before seizure captured - thus poor quality SEEG;  3=grid/strips with seizure; 4=ca-SOZ bilateral and diffuse beyond 1 lobe in each hemisphere; **Etiology Code: 1=FCD or low grade tumor; 2=TSC; 3=non-TSC congenital brain malformation; 4=MTS; 5=acquired brain insult including TBI or prior meningoencephalitis; 6=MRI negative; 7=genetic

# Supplementary Table

S1. Subject Level ROI Description and Modality Subtypes

| ID | ROI | Modality |
| --- | --- | --- |
| 1 | L SFG inf. | 1,2,3 |
|  | L SFG sup | 0,2,3 |
|  | L IFG | 1,0,3 |
|  | L T | 1,0,3 |
| 2 | R Opc | 1,2,3 |
|  | R FP | 0,2,0 |
| 3 | L IFG | 0,2,3 |
|  | RF | 0,2,0 |
|  | RMF | 1,0,3 |
| 4 | R ATS | 1,0,3 |
|  | R MTS | 1,0,0 |
|  | R PTS | 0,2,3 |
|  | R T OPC | 1,0,3 |
| 5 | L AT | 1,2,3 |
|  | R AT | 0,0,3 |
|  | L IFG/PFC | 0,0,3 |
| 6 | R Hipp | 1,0,0 |
|  | R Lat P | 1,2,3 |
|  | R OPC | 0,2,3 |
|  | R OrbF | 0,2,0 |
| 7 | R Post Les | 1,0,0 |
|  | R Ant Med T | 0,0,3 |
|  | L Hipp | 0,2,0 |
| 8 | AML,ALL | 0,2,0 |
|  | IPL | 1,0,0 |
|  | PML,PLL | 1,2,3 |
| 9 | L PFC | 1,0,0 |
|  | R T | 0,2,0 |
|  | R SMG | 0,0,3 |
| 10 | L AT | 0,2,3 |
|  | L Opc | 1,2,3 |
|  | Left Inf T-O | 0,2,3 |
| 11 | ALF/PLF | 1,2,0 |
|  | MMF/LMF | 1,2,0 |
|  | MOrbF/LOrbF | 1,0,0 |
|  | R Hipp | 0,0,3 |
| 12 | R Hipp | 0,0,3 |
|  | R Ant F Opc | 0,2,0 |
|  | R FP | 0,2,3 |
|  | R Ins post | 1,0,0 |
| 13 | R hipp | 1,2,0 |
|  | R Opc/IFG | 0,0,3 |
|  | R post T | 0,0,3 |
| 14 | RAT/R Ins | 1,2,3 |
|  | RPT | 1,2,3 |
|  | R P les | 0,2,3 |
|  | ROrbF | 0,2,0 |
|  | RLF | 0,0,3 |
| 15 | L hipp | 0,2,3 |
|  | R hipp | 0,0,3 |
|  | L PFC lat inf | 1,0,3 |
|  | R PFC | 0,2,3 |
| 16 | Ant Inf Les | 0,2,3 |
|  | Post Inf Les P | 1,0,0 |
|  | AF | 0,0,3 |
| 17 | L post T | 1,2,3 |
|  | R post T | 1,0,3 |
|  | R SMG/P | 0,0,3 |
|  | R O | 1,2,0 |

Subject Level ROI Description and Modality Subtypes. ROIs (regions of interest) were determined based on different modalities: 0 = Regions that were either tested but found inconclusive or excluded from analysis due to lack of clear SOZ localization evidence; 1 = ca-iEEG (clinically annotated iEEG SOZ); 2 = rs-iEEG (resting-state iEEG source-sink index); 3 = rs-fMRI ICA candidate SOZ (independent component analysis-derived regions from resting-state fMRI).**1.2** **Supplementary Figures**


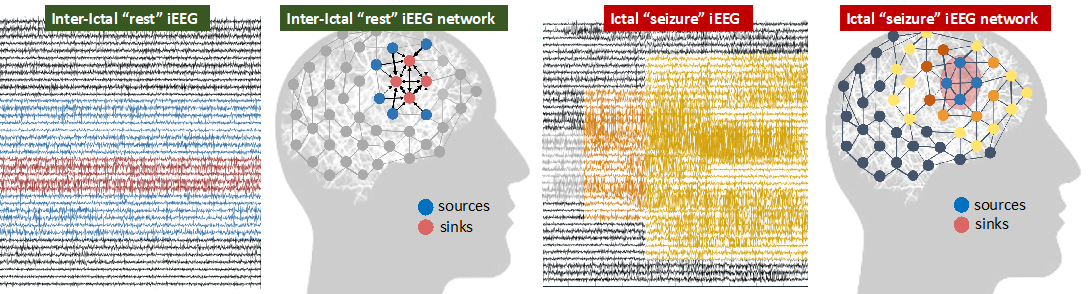
**Figure 1.** Source Sink Hypothesis. A) Interictal (between seizure or at rest) iEEG snapshot and corresponding source-sink schematic where **sinks** represent seizure focus B) Ictal (seizure) EEG snapshot and corresponding source-sink schematic where **sources** represent seizure focus.


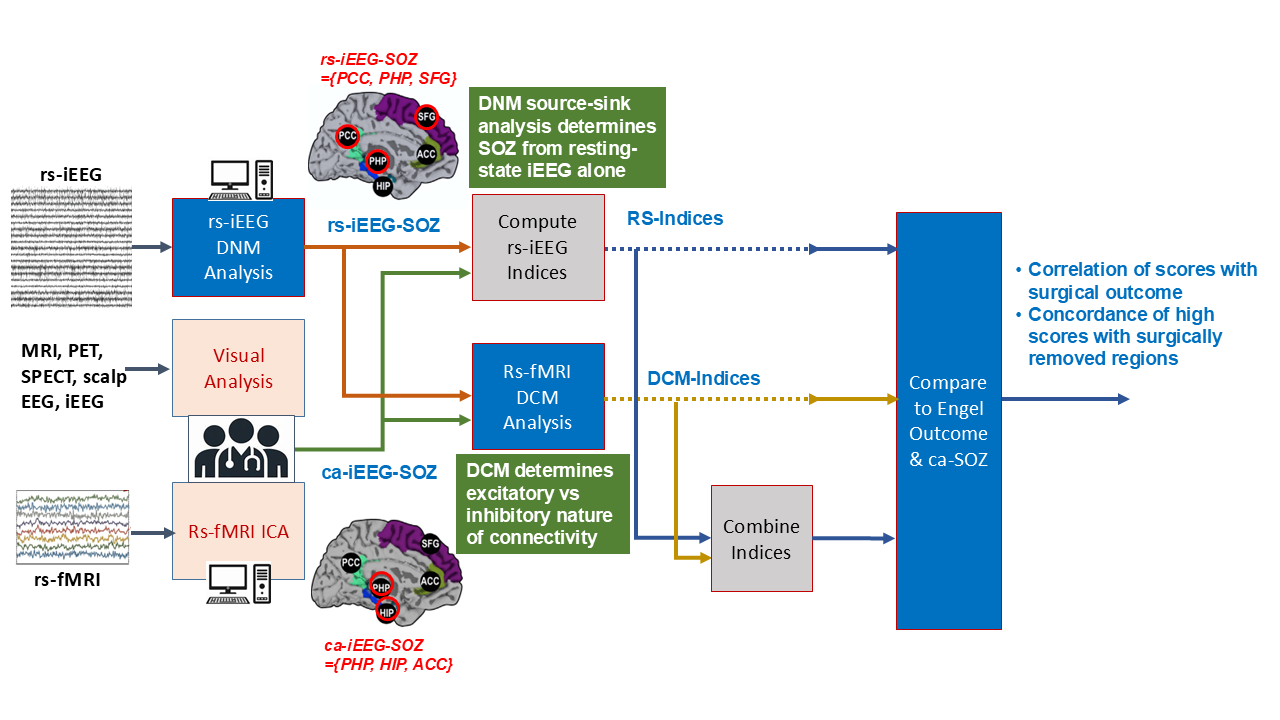
**Figure 2.  Workflow of Study.** Clinically annotated SOZ candidate locations were derived from clinician interpretation of multiple noninvasive modalities including MRI, cvEEG, and rs-fMRI by ICA.  These informed the placement of the stereo electroencephalography (sEEG) depth electrodes.  The clinicians interpreted the sEEG determined the ca-iEEG-SOZ.  From the locations of the depth electrode placement, the rs-fMRI DCM, rs-iEEG DNM, and their combined Dynamic indices were then determined. The indices values were compared to the SOZ determined by clinicians' sEEG interpretation and separately with the combination of surgical location and Engel outcomes.


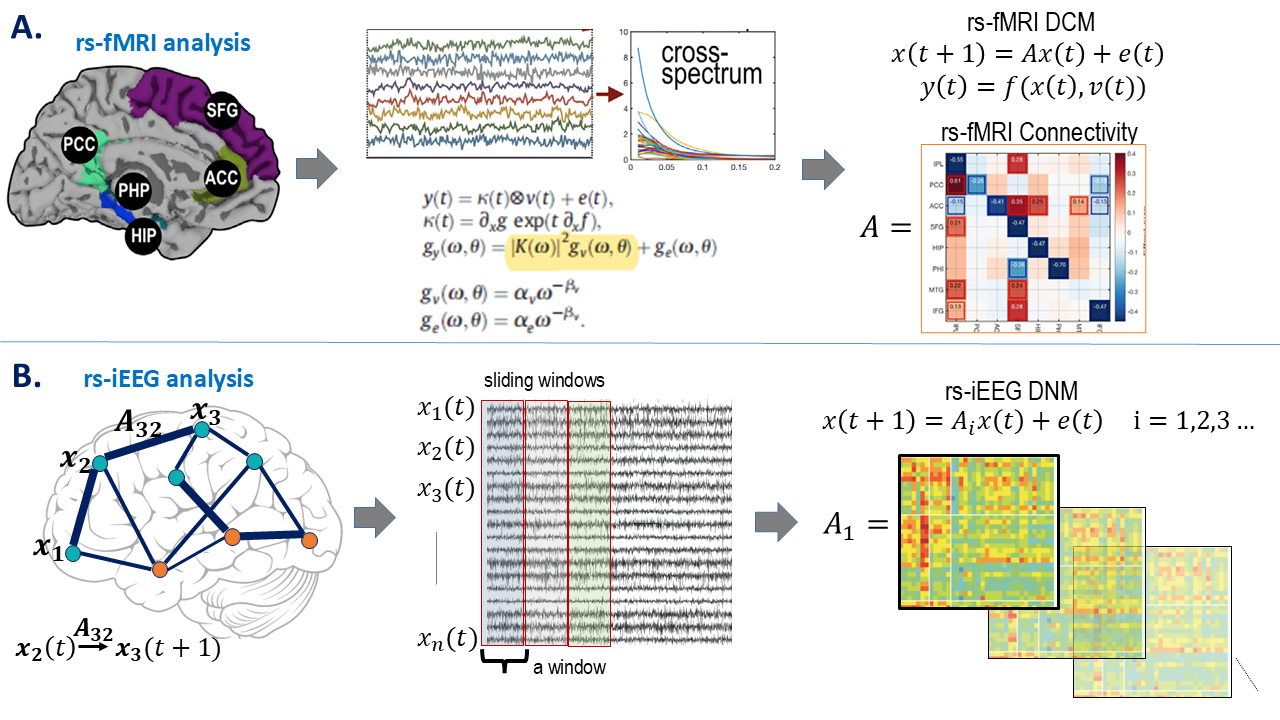


**Figure 3. Model Estimations. A. rs-fMRI model estimation.** Demonstrative node locations with the rs-fMRI BOLD signal over time are transformed from time domain, $y\left( t \right)$, to spectral domain, where $K(\omega)$ is the Fourier transform of the system Volterra kernel $K(t)$, which are a function of the effective connectivity ($A$), and $g_{v}$ represents the effect of other nodes on a given region, and $g_{e}$ is the endogenous effect of the region on itself, and $g_{y}$ is the cross spectrum effective connectivity taking both these exogenous and endogenous signals (amplitude ($\alpha$), slope ($\beta$)). **B. rs-iEEG model estimation.** Model notation defined on electrode implantation. Each channel is a “node” in the iEEG network where the signal on node $i$ is denoted as $x_{i}(t)$. The influence that node $j$has on node $i$ is captured through $A_{ij}$. The DNM based on inter-ictal iEEG is parametrized by the $A$ matrix for each 500-millisecond window, generating a sequence of matrices resulting in one linear time-varying DNM.


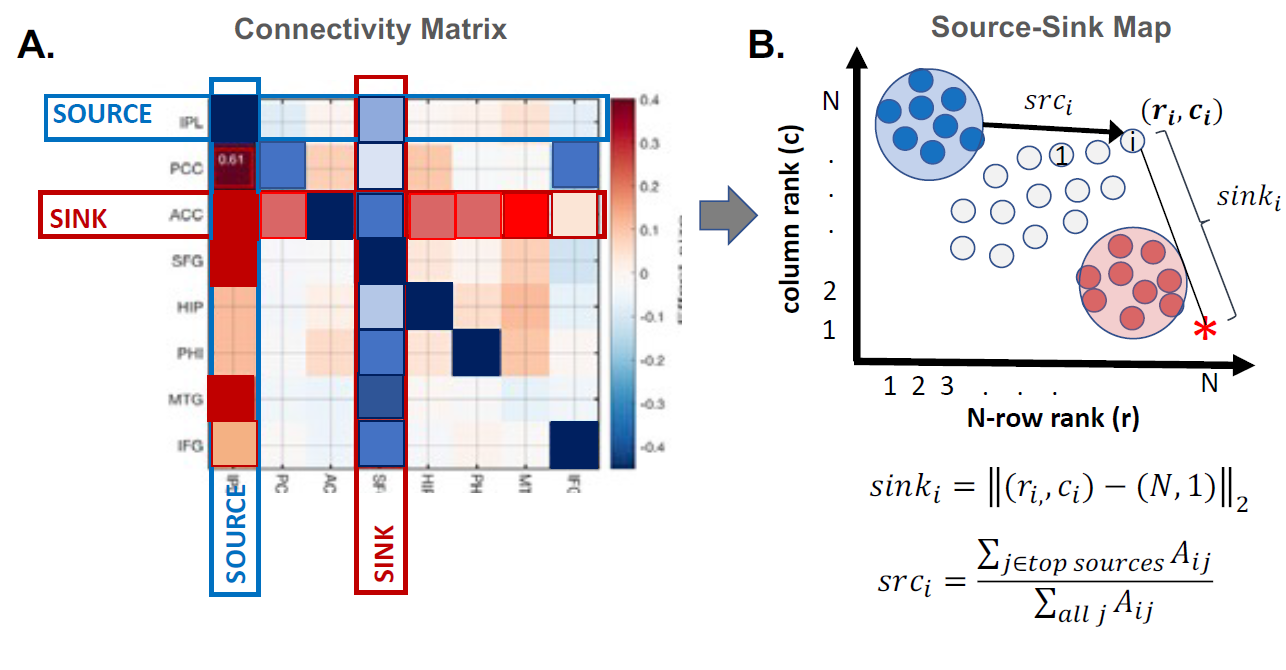
**Figure 4. Patient-Level Source Sink Algorithm.  A.** Example of a connectivity matrix, $A$, derived from either the rs-fMRI DCM or rs-iEEG LTV DNM. Dark blue cells are significantly negative and represent inhibition, while dark red cells are significantly excitatory. If row $j$ is relatively blue and column $j$ is relatively red, node $j$ received inhibition from other nodes in the network and projected excitation to other nodes in the network.  **B.** 2D source-sink map. Pink nodes represent top sinks, blue nodes represent top sources.


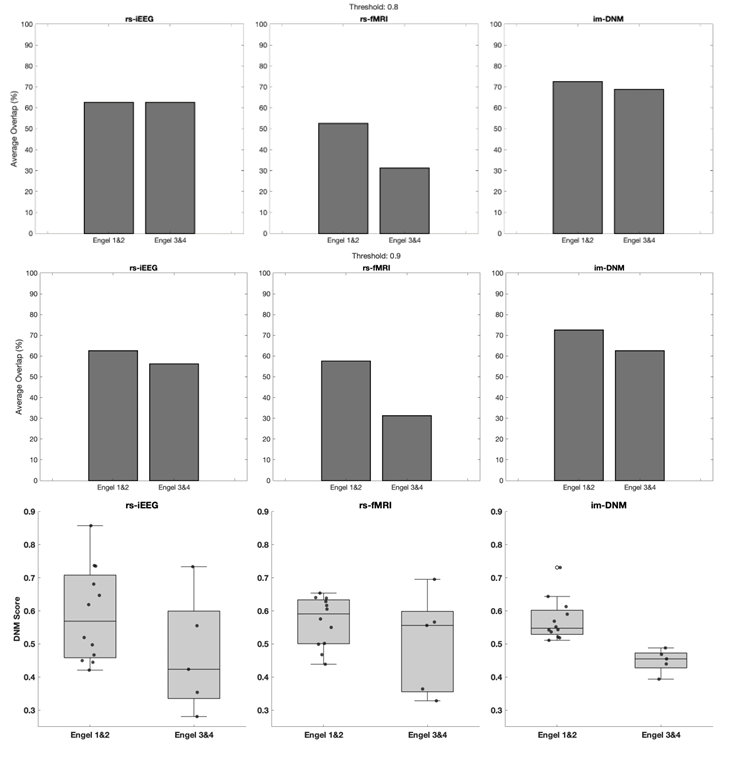


**Figure 5. Comparison of Index Threshold Values for Differentiating Good vs Poor Outcome Groups.**  Left to right are A. rs-iEEG, B. rs-fMRI, and C. combined rs-iEEG and rs-fMRI as im-DNM index results categorized by the patient-level Good vs Poor Surgical Outcomes on the x-axis. The top two rows, indexed as A1-C2, are bar graphs comparing index threshold at 0.8 (top row) and 0.9 (middle row) via the percent of overlap of patients thresholded index values with good vs poor outcomes. Given the lower thresholded index value overlap with the higher threshold, the bottom row, A3-C3, of box plots (thresholded at 0.9) compares distributions of patient-level outcomes by biomarker subtype, showing the combined index im-DNM with the optimal outcome group differentiation. The regions evaluated do not necessarily correspond with the surgically targeted region, as the goal was to evaluate regions with clinically reasoned SOZ hypothesis, and several patients had poor surgical outcome, implying the SOZ was outside the resected region.
